# Electron microscopic and preparative methods for the analysis of isopod cuticle

**DOI:** 10.3897/zookeys.176.2294

**Published:** 2012-03-20

**Authors:** Bastian H. M. Seidl, Andreas Ziegler

**Affiliations:** 1 Central Facility for Electron Microscopy, University of Ulm, Albert-Einstein-Allee 11, 89069 Ulm, Germany

**Keywords:** Isopoda, cuticle, ultrastructure, *Porcellio scaber*

## Abstract

The crustacean cuticle consists of a complex organic matrix and a mineral phase. The physical and chemical properties of the cuticle are corellated to the specific functions of cuticular elements, leading to a large variety in its structure and composition. Investigation of the structure-function relationship in crustacean cuticle requires sophisticated methodological tools for the analysis of different aspects of the cuticular architecture. In the present paper we report improved preparation methods that, in combination with various electron microscopic techniques, have led to new insights of cuticle structure and composition in the tergite cuticle of *Porcellio scaber*. We used thin sections of non-decalcified tergites and decalcified resin embedded material for transmission electron microscopy and scanning transmission electron microscopy. Etched sagittal planes of bulk tergite samples were analysed with field emission scanning electron microscopy. We have found a distinct distal region within the exocuticle that differs from the subjacent proximal exocuticle in the arrangement of fibres. Within this distal exocuticle chitin-protein fibrils assemble to fibres with diameters between 15 and 50 nm that are embedded in a mineral matrix. In the proximal exocuticle and the endocuticle fibrils do not assemble to fibres and are surrounded by mineral individually. Furthermore, we show that the pore canals are filled with mineral, and demonstrate that mild etching of polished sagittal cuticle surfaces reveals regions containing mineral of diverse solubility.

## Introduction

The structural organisation of the crustacean cuticle has recently led to increasing interest because of its high structural and chemical variability. Furthermore, the outstanding mechanical properties of the crustacean cuticle have attracted attention to its high potential for biomimetic technical applications. The organic matrix of the arthropod cuticle is hierarchically organised (Vincent 2002, Nikolov et al. 2011). Chitin chains form crystalline alpha chitin fibrils (Carlström 1957) that are surrounded by protein (Blackwell and Weih 1980). These chitin-protein fibrils assemble to larger fibres that form planes in which all fibres are orientated in the same direction. Recent studies have shown, that in some species these planes are formed by fibrils that are not assembled to fibres (Nicolov et al. 2011, Seidl et al. 2011). Many of these planes are stacked upon another in a way that the direction of the fibrils/fibres is twisted by a small angle against that of the preceding plane, thereby forming a twisted plywood structure (Bouligand 1972). In slightly oblique transversal sections through the cuticle, the twisted plywood structure results in a virtual stratification marked by chitin-protein fibrils/fibres that are oriented parallel to the cutting plane (Giraud-Guille 1984). The turn of fibrils/fibres by 180 degrees defines one stack of planes. The distance between two planes with parallel fibrils/fibres is called stacking height. These stacks form three of the four principal layers of the cuticle: the exocuticle, the endocuticle and the innermost membranous layer. The epicuticle is the outermost principal layer and contains no chitin-protein fibrils or fibres but proteins and waxy layers that serve as a barrier for water loss in terrestrial arthropods (Compère 1990). In crustaceans the exocuticle and endocuticle are mineralised by calcite, amorphous calcium carbonate (ACC), and to some degree by amorphous calcium phosphate (Dillaman et al. 2005, Boßelmann et al. 2007, Neues et al. 2007, Hild et al. 2008, Neues et al. 2011).

The crustacean cuticle is an exoskeleton that provides support, protection against predation and environmental strains, sites for muscle attachment, and structures involved in receiving sensory information. The cuticle surrounds the whole animal and is subdivided into skeletal elements such as segments of the main body and legs, claws, mouthparts, eye lenses, etc. Flexible cuticular membranes often connect skeletal elements to allow for relative movements. The structure and composition of the cuticle are adapted to the function of a specific skeletal element and to the habitat of the animal (Neues et al. 2007, Hild et al. 2009). This results in a large variety of cuticle structure and composition requiring a comparative approach to understand the structure-function-composition relationship of crustacean cuticle.

In the present paper we describe improved preparation methods for the cuticle of terrestrial isopods using the tergite cuticle of *Porcellio scaber* as an experimental model. The cuticle of *Porcellio scaber* and other related species has been studied previously (Price and Holdich 1980, Compère 1990, Štrus and Compère 1996, Štrus and Blejec 2001, Hild et al. 2008, 2009, Huber and Ziegler 2011, Mrak et al. 2011, Ruangchai et al. 2011, Seidl et al. 2011). Our results confirm most structural features found earlier and have led to new insights in the ultrastructure and composition of the tergite cuticle.

## Methods

*Porcellio scaber*Latreille, 1804 were collected from biotopes near Ulm, Germany, kept in plastic containers filled with soil and bark, and fed with fresh potatoes and dry oak leaves. Animals in the intermoult stage were identified by the lack of external signs of moulting such as sternal CaCO_3_ deposits. The intermoult stage was further verified during tergite sample preparation and structural analysis. Specimens with any signs of apolysis and samples with soft or incompletely developed cuticle were not used. We used the tergites of pereonites 2–7.

For transmission and low voltage scanning transmission electron microscopy (TEM, STEM) of the organic phase, in decalcified EPON resin embedded samples, dissection of the cuticle was performed in a decalcification/fixation solution containing 0.05 mol L^-1^ EDTA for decalcification, 2.5% glutaraldehyde and 2% paraformaldehyde for fixation of organic material, and 0.25 mol L^-1^ HEPES (pH 7.8) for buffering pH during decalcification. The samples were incubated in fresh solution for 34 days at 4 °C. During simultaneous decalcification and fixation, parts of the organic matrix, which emerge slowly from surrounding mineral and are thus exposed to the solution, are immediately fixed by the aldehyde. The decalcified tergites were then washed 3 times in bi-distilled H_2_O for 10 minutes each and postfixed for 1 h in a solution containing 1% OsO_4_ and 0.8% K_4_[Fe(CN)_6_] (White et al. 1979). After postfixing, they were washed again 3 times in bi-distilled H_2_O for 10 minutes each and dehydrated in a graded series of isopropanol. Part of the samples was block contrasted in a solution of 0.5% uranyl acetate in denatured ethanol for 30 minutes, followed by 3 washing steps in ethanol for 4 minutes each. Then the samples were washed two times in acetone for approximately 4 minutes each and embedded in EPON resin. Sagittal, 50 nm thick sections of the embedded tergites were cut using an Ultracut ultramicrotome (Leica). The sections were placed on 300 mesh copper grids and analysed using a Jeol 1400 TEM at 80 kV acceleration voltage or with a Hitachi S-5200 field emission SEM (FE-SEM) operated at 30 kV.

STEM is a method where the electron beam is focused in a spot which is raster scanned across a sample. We used a high-resolution FE-SEM equipped with a dark field STEM-detector to perform STEM analysis at low voltages (30 kV), which considerably enhances contrast and still yields spatial resolution of about 1 nm. The contrast arises from local density of the sample that is in particular useful for the analysis of unstained sections of non-demineralised material.

For microscopy of non-decalcified samples, dissection in aqueous solutions should be avoided because this would lead to dissolution of the mineral phase within the endocuticle. *In vitro* experiments have demonstrated that the ACC polymorph is ten times more soluble than its crystalline counterparts (Brečevic and Nielsen 1989), and it has been shown that the endocuticle of isopod cuticle contains ACC as the main mineral component. Although ACC is stabilised in biological tissues, preliminary experiments have shown that it quickly dissolves in aqueous solutions even at strongly basic pH values. Therefore, we used 100% methanol for dissection, in which ACC remains stable for at least one month (Becker et al. 2003). After dissection samples were washed in bi-distilled water for 1–2 seconds to remove tissue saline at the surface and then for 25 seconds in 100% methanol to remove water. The specimens were left to air dry at room temperature (Hild et al. 2009).

To obtain polished sagittal faces and sections of native (non-demineralised) cuticle, tergites were first glued to cylindrical aluminium holders (diameter 3 mm) using super glue gel. Sagittal planes were cut with a glass knife and then polished with a Diatome diamond knife according to the method described previously (Fabritius et al. 2005). A Diatome ultra 35° diamond knife and a Diatome static line II ionisator were used to cut dry 60 nm thick sections. The sections were transferred to carbon coated formvar film on 300 mesh copper grids using an eyelash. To flatten the sections, another carbon coated formvar film on a 300 mesh copper grid was placed onto the grid carrying the sections. Sections were flattened by the weight of a brass-rod 3 mm in diameter and approximately 200 mm long with a polished end-surface. Finally, sections were coated with a 4–8 nm thick layer of carbon using a BAF 300 freeze-etch device (Balzers) and analysed by low voltage STEM (Hitachi S-5200 at 30 kV).

To expose the organic matrix in non-decalcified tergite cuticle, samples were first polished as described above and then etched for 20 seconds with an aqueous solution of 2.5% glutaraldehyde and 0.01 mol L^-1^ MOPS buffer adjusted to pH 6.5 or 0.1 mol L^-1^ HEPES buffer adjusted to 8.0. Then, the samples were washed 3 times for 10 minutes with isopropanol, critical point dried, rotary shadowed with 4 nm of platinum (BAF 300, Balzers) and analysed with a Hitachi S-5200 FE-SEM at 4 kV using the secondary electron detector. Secondary electrons (SE) have a low energy and thus only those elicited from near the sample surface can reach the detector and can thus contribute to imaging of the surface topography. A SEM with a field emission gun was used to obtain high resolution (nominally 1.8 to 0.5 nm at 1 to 30 kV, respectively) at low acceleration voltages as a result of the small diameter of the focused electron beam (spot size).

## Results

### Decalcified thin sections

**Figure 1.**
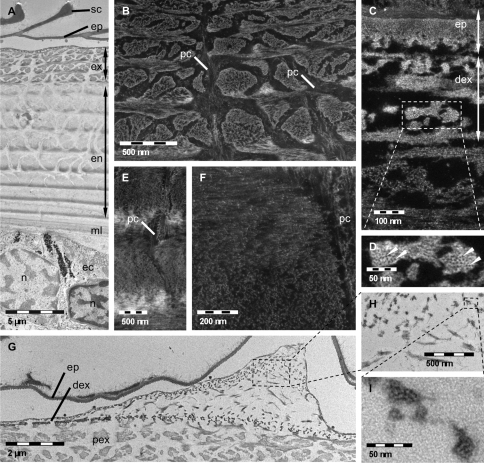
TEM **A, G, H, I** and STEM **B, C, D, E, F** micrographs of decalcified and EPON embedded tergites of *Porcellio scaber*. **A** Sagittal overview showing epicuticle (ep), exocuticle (ex), endocuticle (en) and membranous layer (ml), ec, epithelial cell; n, nucleus; sc, epicuticular scale. **B** Proximal exocuticle. Dense network of pore canals (pc) containing fibrils or fibres following the direction of the pore canal. **C, D** Fibres of the distal exocuticle (dex) consisting of approximately 3 nm thick unstained chitin crystallites (arrowheads) surrounded by densely stained proteins. **E, F** In the endocuticle single fibrils form the twisted plywood structure. The pore canals contain vertical fibrils or fibres. **G, H, I** Section through a cuticular thickening. The increase in cuticle thickness is brought about by an increase of the stacking height in the distal exocuticle only. **H, I** Details of **G** confirm the typical structure of fibres within the distal exocuticle.

TEM and STEM analyses of simultaneously decalcified and fixed samples provide detailed information about the specific hierarchical organisation of the organic matrix of the cuticle from its principal layers down to single protein fibrils and chitin crystallites. Exo- and endocuticle can be clearly distinguished by differences in contrast and morphology (Figure 1). The organic matrix of the exocuticle is more intensely stained than the endocuticle one, resulting in a sharp border between them (Figure 1A). In the proximal exocuticle pore canals are more branched than in the endocuticle and a high variation in the width of pore canals can be observed, probably due to twisting of spindle-shaped pore canals. In sagittal sections, this leads to an islelike appearance of the part of the organic matrix that is organised in a twisted plywood structure (Figures 1A, B). In the sagittal sections of the endocuticle however, pore canals have a lunulate appearance. The width of the pore canals decreases towards proximal stacks (Figure 1A). There is a clear difference in the organisation at the level of fibrils and fibres between a distinct distal and the proximal region of the exocuticle. Within the distal region of the exocuticle chitin-protein fibrils form fibres consisting of electronlucent approximately 3 nm thick chitin crystallites surrounded by densely stained protein (Figures 1C, D). The fibres vary in size and shape. Most fibres have a diameter of approximately 25 nm but fibres with diameters up to 50 nm also appear regularly. Within the proximal part of the exocuticle as well as in the endocuticle, fibrils appear not to form fibres (Figures 1E, F). Local thickenings of the cuticle, regularly observed in thin sections and probably representing edges of tubercles, derive from an increase in the height of stacks in the distal exocuticle, whereas the thickness of stacks of the endocuticle remains unchanged (Figures 1G–I). The 150 nm thick epicuticle consisting of an electron-lucent outer and an electron-dense inner epicuticle (Figure 1C) remains unaltered as well (Figure 1G).

### Non-decalcified thin sections

**Figure 2.**
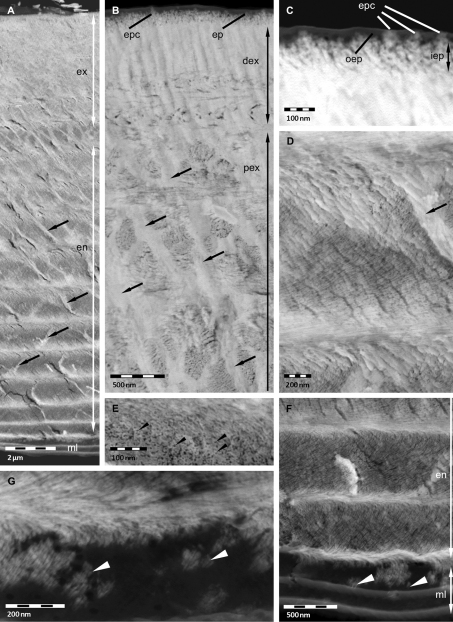
STEM micrographs of non-decalcified tergites of *Porcellio scaber*. **A** Overview showing the mineralised exocuticle (ex) and endocuticle en and the unmineralised membranous layer (ml). The pore canals (arrows) are mineralised. **B, C** Exocuticle and epicuticle (ep). Approximately 25 nm thick fibres in the distal exocuticle (dex) and approximately 6 nm thick fibrils in the proximal exocuticle (pex). Pore canals arrows contain mineral. The inner epicuticle (iep) appears partly mineralised, and the outer epicuticle (oep) unmineralised, except epicuticular pore canals (epc). **D, E** Endocuticle with mineralised pore canal (arrow). Approximately 6 nm thick chitin-protein fibrils (black arrowheads) individually surrounded by mineral forming a twisted plywood structure. **F, G** Single mineral rods (white arrowheads) at the border between membranous layer and endocuticle.

Thin sections of non-decalcified cuticle are of value for studying the distribution of mineral and its spatial relation to the organic phase. Figure 2A shows a sagittal section through the whole cuticle. We confirm that the membranous layer and most regions of the outer epicuticle are devoid of mineral (Figures 2A, C). It appears that parts of the inner epicuticle and perhaps epicuticular pore canals may well contain mineral (Figures 2B, C). It is of interest that within the whole exo- and endocuticle pore canals are filled with mineral (Figures 2A, B, D). Thin pore canals within proximal stacks of the endocuticle can well be seen (Figure 2A). In the 11.5 µm thick distal exocuticle mineral surrounds electron-lucent organic fibres approximately 25 nm in diameter indicating that the proteinaceous material around the chitin crystallites shown in Figures 1C and 1D remains unmineralised (Figure 2B). Within the proximal exocuticle and the whole endocuticle, we observe electron-lucent structures with diameters of approximately 6 nm, corresponding to single chitin-protein fibrils (Figures 2D, E). They are surrounded by mineral that forms interconnected tube-like structures, confirming that in these layers the fibrils do not assemble to fibres. At the border between the endocuticle and the membranous layer rod-like mineral structures occur in a partly mineralised region (Figures 2F, G).

### Etched surface samples

**Figure 3.**
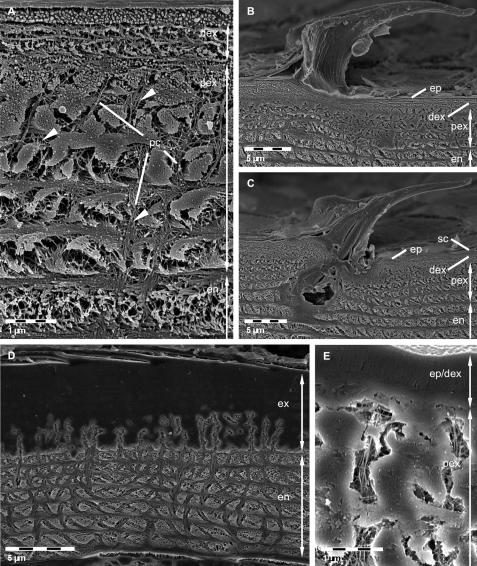
FE-SEM micrographs of polished sagittal plane through bulk tergite samples etched at pH 6.5 **A, B, C** and 8.0 **D, E**. **A** Fibres in the distal exocuticle (dex) and the isle-like structure of the proximal exocuticle (pex) caused by large pore canals (pc). Fibrils or fibres (arrowheads) in the pore canals are well visible. en, endocuticle. **B, C** Side views of tricorn sensilla. The epicuticular unmineralised material forming the sensilla is well distinguishable from the mineralised exo- and endocuticle. ep, epicuticle; sc, epicuticular scale. **D, E** Mild etching reveals regions containing mineral of different solubility. Mineral within the endocuticle appears etched whereas most regions within the exocuticle (ex) remain unaltered. Note etching within pore canals of the exocuticle.

Etched surfaces are in particular suitable to visualize the distribution of organic fibrils or fibres within the pore canal system. In addition, because shrinkage due to demineralisation followed by drying of the cuticle can be minimised, some quantitative aspects of the cuticle, such as the stacking height and thickness of exo- and endocuticle can be measured more accurately. Just as in non-decalcified thin sections the distal exocuticle is approximately 1 µm thick. Fibres within the pore canals that are only faintly visible in TEM sections can be well observed (Figure 3A). Etching of polished samples can also be used to distinguish mineralised from non-mineralised structures. A side view of a tricorn sensillum is shown in Figure 3B. Non-mineralised epicuticular structures in the cutting plane can well be distinguished from surrounding mineralised cuticular layers (Figures 3B, C). Depending on the pH used for etching polished and etched cuticle surfaces can be used to discern regions differing in the solubility of the mineral phases e.g. between ACC and calcite. Etched at pH 6.5, fibres or fibrils in all principal layers are visible (Figures 3A–C). At pH 8.0, mineral was etched away in the endocuticle and partly in the exocuticle (Figure 3D). In the latter etching occurs mostly in proximal regions of the pore canals, sometimes also more distally, but not in the distal exocuticle (Figures 3D, E).

## Discussion

Using improved preparation methods for conventional TEM, low voltage STEM and FESEM, we can confirm all general features of the tergite cuticle described in several previous publications on the integument cuticle of terrestrial isopods (Price and Holdich 1980, Štrus and Compère 1996, Ziegler 1997, Štrus and Blejec 2001). In addition, we describe features that have not been described previously in the cuticle of *Porcellio scaber*, such as the presence of a distinct distal exocuticle that differs from the subjacent proximal exocuticle, pore canals that contain mineral, the spatial arrangement of mineral around fibres within the distal exocuticle versus mineral around fibrils in the proximal exocuticle and endocuticle, and the distribution of well soluble mineral phases.

Simultaneous decalcification and fixing of samples allows for visualising the structure on fibre level within the distal exocuticle such as chitin crystallites embedded in a proteinaceous matrix. In *Porcellio scaber* these fibres have the same structure as those reported previously for the distal exocuticle in the tergites of *Tylos europaeus* (Seidl et al. 2011). Interestingly, the relative size of distal and proximal exocuticle differs between these species. In *Tylos europaeus*, the distal exocuticle is between 4 and 10 µm thick and consists of at least 4 stacks versus 11.5 µm thick and just 12 stacks in *Porcellio scaber*. In reverse, the proximal exocuticle consists of 2 stacks in *Tylos europaeus* and 4 stacksin *Porcellio scaber*. The distal exocuticle appears to play a specific role in the formation of local thickenings in the tergites of *Porcellio scaber* (present study) and the formation of micro-tubercles in *Tylos europaeus* (Seidl et al. 2011), in which thickening of the cuticle occurs by an increase in the height of the stacks in the distal exocuticle.

Studies on the distribution of calcite and ACC within the cuticle of three terrestrial isopods using Raman spectroscopy and Raman imaging techniques (Hild et al. 2008, 2009) have shown that calcite occurs within the exocuticle with little ACC in the proximal region, whereas the endocuticle contains ACC only. Because of the high solubility of ACC (Brecevic and Nielsen 1989) versus calcite at pH 8.0, the difference in etching of the surfaces on polished exo- and endocuticle confirms that only the exocuticle contains calcite. In regions of the exocuticle containing well soluble mineral phases such as ACC, etching exposes pore canals that can be identified by their vertically oriented fibrils or fibres. This suggests that ACC within the exocuticle is mostly localized within pore canals.

Previous structural analysis of broken non-demineralised cuticle revealed two regions in the exocuticle of *Porcellio scaber*, *Armadillidium vulgare*, *Titanethes albus* und *Tylos europaeus*: a distal region in which broken faces appear smooth, hardly exposing any organic fibres (smooth layer), and a subjacent exocuticular layer in which stacks of mineralised twisted fibrils are well visible (Hild et al. 2008, 2009, Seidl et al. 2011). It is of particular interest that, according to the present results and those on the Tylidae *Tylos europaeus*, the thickness of the distal exocuticle appears to correlate with the smooth layer, although in this species peculiar polyhedral textures occur in addition to the smooth regions. Furthermore, we recently confirmed this correlation for the Tylidae *Helleria brevicornis* (Seidl and Ziegler 2011). Together, these observationssuggest that the organisation of the organic matrix at the level of fibres and fibrils may influence the fracture behaviour of exocuticular layers. This is in accordance with recent results on multi-scale modelling of crustacean cuticle that stress the importance of structural properties on the mechanical behaviour at all hierarchical levels including those at the microscale (Nikolov et al. 2010, 2011, Fabritius et al. 2011).

According to the model for insect cuticle, proteins form a helicoidal sheath around 19 anti-parallel chitin chains that form the approximately 3 nm thick crystallites (Blackwell and Weih 1980, Neville 1998, Vincent and Wegst 2004). The thickness of organic fibrils within non-decalcified thin sections of the endocuticle and proximal exocuticle presented here is in good agreement with the size of chitin-protein fibrils in the insect model. Thus it appears that in these layers single fibrils are surrounded by mineral and do not assemble to fibres, in contrast to the organic matrix of the distal exocuticle in which bundles of chitin-protein fibrils (fibres) are surrounded by mineral. These results accord well with previous results on the tergite cuticle of *Tylos europaeus* (Seidl et al. 2011). The mineralisation at the level of chitin-protein fibrils has also been described for the cuticle of *Homarus americanus* (Nikolov et al. 2011).

Other important structural features of the crustacean cuticle are the distribution, shape and size of pore canals. The pore canal system serves not only as a transport system during cuticle mineralisation (Travis 1965, Roer 1980, Giraud-Guille 1984, Roer and Dillaman 1984) but can also fulfil mechanical tasks (Sachs et al. 2008). In contrast to some decapod species in which pore canals are devoid of mineral (Fabritius et al. 2011), the pore canals of *Porcellio scaber* as well as *Tylos europaeus* are filled with mineral. This is certainly affecting the mechanical properties of the cuticle, probably leading to higher cuticle stability compared to cuticles with pore canals that contain no mineral.

## Conclusion

The present paper shows that investigation of crustacean cuticle by using an improved protocol for demineralisation and fixation for TEM/STEM, the analysis of thin sections of non-demineralised cuticle, and etching of polished cuticle samples can reveal new aspects of cuticular structure and mineral distribution. Combining the results from decalcified resin embedded specimens and those from thin sections of non-decalcified cuticle has led to the demonstration of a distinct distal exocuticle that differs in the organisation of the organic matrix at the level of chitin-protein fibrils and fibres, from those in the proximal exocuticle and the whole endocuticle. Thin sectioning of non-decalcified cuticle samples in combination with low voltage STEM is also a valuable method to gain new and detailed information about the distribution of mineral and organic fibrils/fibreswith high spatial resolution. In combination with TEM/STEM of resin embedded material, this method revealed details such as mineral filled pore canals and chitin-protein fibrils forming a twisted plywood structure without assembling in fibres. Etching of polished samples has the advantage that shrinkage of mineralised regions is minimised. This is of particular interest when length scales have to be quantified with high accuracy. Furthermore, etching reveals well the orientation of fibrous organic structures that, in combination with differential etching of amorphous and crystalline mineral phases, allows for the allocation of mineral phases to specific cuticular structures.
